# A Potential Risk of Overestimating Apparent Diffusion Coefficient in Parotid Glands

**DOI:** 10.1371/journal.pone.0124118

**Published:** 2015-04-29

**Authors:** Yi-Jui Liu, Yi-Hsiung Lee, Hing-Chiu Chang, Teng-Yi Huang, Hui-Chu Chiu, Chih-Wei Wang, Ta-Wei Chiou, Kang Hsu, Chun-Jung Juan, Guo-Shu Huang, Hsian-He Hsu

**Affiliations:** 1 Department of Automatic Control Engineering, Feng Chia University, Taichung, Taiwan, Republic of China; 2 Ph.D. program in Electrical and Communication Engineering in Feng Chia University, Taichung, Taiwan, Republic of China; 3 Brain Imaging and Analysis Center, Duke University Medical Center, Durham, North Carolina, United States of America; 4 Applied Science Laboratory, GE Healthcare, Taipei, Taiwan; 5 Department of Electrical Engineering, National Taiwan University of Science and Technology, Taipei, Taiwan, Republic of China; 6 Ph.D. program of Technology Management, Chung Hua University, Hsinchu, Taiwan, Republic of China; 7 Department of Radiology, National Defense Medical Center, Taipei, Taiwan, Republic of China; 8 Department of Radiology, Tri-Service General Hospital, Taipei, Taiwan, Republic of China; 9 Department of Medicine, Taipei Medical University, Taipei, Taiwan, Republic of China; 10 Department of Dentistry, National Defense Medical Center, Taipei, Taiwan, Republic of China; University of Maryland, College Park, UNITED STATES

## Abstract

**Objectives:**

To investigate transient signal loss on diffusion weighted images (DWI) and overestimation of apparent diffusion coefficient (ADC) in parotid glands using single shot echoplanar DWI (EPDWI).

**Materials and Methods:**

This study enrolled 6 healthy subjects and 7 patients receiving radiotherapy. All participants received dynamic EPDWI with a total of 8 repetitions. Imaging quality of DWI was evaluated. Probability of severe overestimation of ADC (soADC), defined by an ADC ratio more than 1.2, was calculated. Error on T2WI, DWI, and ADC was computed. Statistical analysis included paired Student t testing and Mann-Whitney U test. A P value less than 0.05 was considered statistically significant.

**Results:**

Transient signal loss was visually detected on some excitations of DWI but not on T2WI or mean DWI. soADC occurred randomly among 8 excitations and 3 directions of diffusion encoding gradients. Probability of soADC was significantly higher in radiotherapy group (42.86%) than in healthy group (24.39%). The mean error percentage decreased as the number of excitations increased on all images, and, it was smallest on T2WI, followed by DWI and ADC in an increasing order.

**Conclusions:**

Transient signal loss on DWI was successfully detected by dynamic EPDWI. The signal loss on DWI and overestimation of ADC could be partially remedied by increasing the number of excitations.

## Introduction

Over the last two decades, diffusion-weighted imaging (DWI) has been increasingly applied to characterize the diffusivity of tissue in head and neck by measuring apparent diffusion coefficient (ADC). Accurate and robust measurement of ADC is of paramount importance because it has been used to differentiate malignant from benign tumors [1], to distinguish malignant lymphomas from carcinomas [2], and to monitor treatment response of squamous cell carcinoma of head and neck [3].

Unfortunately, there is a wide variation regarding ADC measurements in head and neck. Taking parotid glands as an example, the ADC measured in different studies varies widely from as low as 0.28 × 10^–3^ mm^2^/s [4] to as high as 2.46 × 10^–3^ mm^2^/s [5] in healthy subjects. Such a wide range of ADC has exceeded the difference of ADC among different tumors and the change of ADC before and after treatment of tumors. In addition, direct comparison of parotid ADC in different study groups becomes difficult due to several technical factors that cause the discrepancy in parotid ADC measurements, including strength and number of diffusion gradients (b values) [6], pulse sequences [7, 8], and acceleration factors [7].

Single shot echoplanar diffusion-weighted image (EPDWI) is the pulse sequence most commonly used to measure ADC in head and neck thanks to the merit of its fast data acquisition. However, EPDWI is susceptible to magnetic susceptibility artifacts related to metal materials and air-soft tissue interfaces [7]. It is plausible that EPDWI might also be vulnerable to transient signal loss originating from intravoxel phase dispersion possibly induced by subject motion. The purpose of this study was to investigate the transient signal loss on DWI and overestimation of ADC in parotid glands using EPDWI.

## Materials and Methods

### Subjects

This study was approved by the institution review board of Tri-Service General Hospital. Written informed consents were obtained from all participants recruited. A total of 13 subjects were enrolled, including 6 healthy volunteers (mean age 33 years, 5 men and 1 woman) and 7 patients (mean age 57 years, 6 men and 1 woman) with radiotherapy to the head and neck. All subjects were free from parotid lesions.

### MR Imaging Protocol

All subjects received EPDWI on one 1.5T clinical MR scanner (Signa HDx, GE Healthcare, Milwaukee, WI) using an eight-channel neurovascular head and neck phase array coil. Gradient-echo MR imaging was first used to acquire three-plane orthogonal images to identify the location of parotid glands, after which the diffusion-weighted imaging scans were performed.

**Fig 1:**
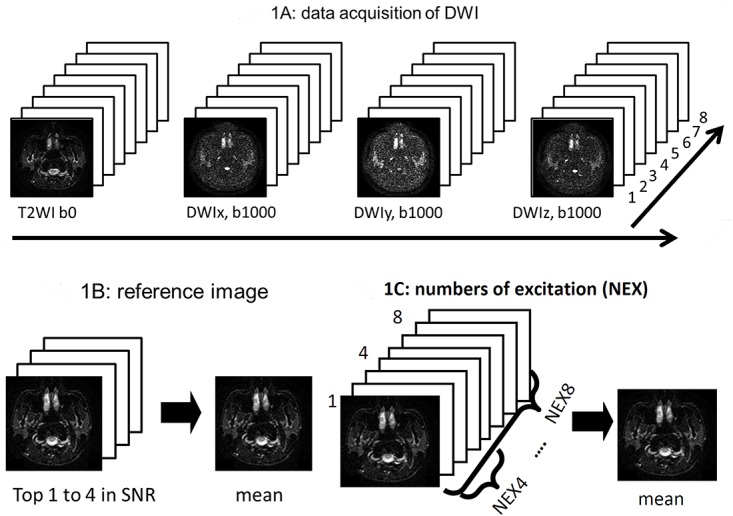
Data acquisition and image processing. (A) The first excitation of diffusion-weighted images including T2WI, DWIx, DWIy and DWIz was discarded as a dummy scan (not shown here). After reaching the equilibrium state of the magnetization, diffusion-weighted images were dynamically acquired in the order of T2WI, DWIx, DWIy and DWIz (long arrow) on each excitation for a total of 8 repetitions (short arrow). (B) Images with the top four SNR were averaged to generate a reference image. By this way, mean T2WI, DWI_(x, y and z)_ were generated to serve as reference images. Then reference ADC_(x, y and z)_ were calculated based on the reference T2WI and DWI_(x, y and z)_. (C) T2WI, DWI_(x, y and z)_ and ADC_(x, y and z)_ with a NEX of 3 to 8 were generated from the raw images. For example, a set of “3” continuous raw images were averaged to generate an image with a NEX = 3, and so forth.

Single shot EPDWIs were acquired with diffusion-encoding gradients (b = 0 s/mm^2^ for a T2-weighted image and 1000 s/mm^2^ along three orthogonal directions) applied. The scan parameters were: TR/TE/flip angle = 4000 ms/95 ms/90°, field of view = 240 × 240 mm, matrix size = 128 × 128 interpolated to 256 × 256, slice thickness = 5 mm. A total of 17 slices were scanned to cover from the level of skull base to the level of submandibular glands. A total of nine excitations were consecutively acquired with each excitation comprising one T2WI (b = 0 s/mm^2^) and three DWIs (b = 1000 s/mm^2^), including DWIx, DWIy and DWIz (Fig 1A). The total scan time for DWI acquisition was 2:34.

### Image Processing and Data Analysis

After discarding data from the first excitation, which was regarded as dummy scan, all other raw data stored in DICOM format were digitally transferred from the MR scanner to a personal computer for post-processing. The post-processing software was developed in-house (by L.Y.J & L.Y.H.) using MATLAB (MathWorks, Natick, MA). ADC maps were computed from T2WI and DWI images along each direction on each excitation. ADC derivation was based on the Stejskal-Tanner relationship: ADC = ln[S_*b = 1000*_ / S_*b = 0*_]/(-*b*), where S_*b = 1000*_ and S_*b = 0*_ are signal intensities of images with b value of 1000 s/mm^2^ and 0 s/mm^2^, respectively.

Imaging quality of DWI was evaluated by two authors (Y.S.L. and C.J.J.) in consensus. One single author (Y.S.L.) drew the region-of-interest (ROI) for parotid glands slice-by-slice on the mean DWI. All ROIs were verified by a neuroradiologist (C.J.J.) with ten years of experience in head-and-neck imaging interpretation. Signal-to-noise ratio (SNR) of each ROI on each scan was computed. For comparison, T2WI, DWIx, DWIy, and DWIz averaged from the four highest SNR data sets were regarded as reference images, from which the reference ADCx, ADCy, and ADCz were generated (Fig 1B).

By dividing the ADC on each excitation by the ADC on reference image, ADC ratio was calculated on a pixel-by-pixel basis. ADC ratio was used to monitor the variation of ADC and to evaluate the probability of severe overestimation of ADC (soADC) on each excitation. soADC was defined as a mean ADC ratio more than 1.2. Probability of soADC, defined as the number of ROIs with soADC divided by the number of all ROIs, was calculated.

Then, n excitations of T2WI, DWI_(x, y and z)_ and ADC_(x, y and z)_ were averaged, respectively, with n stepping from 3 to 8 (Fig 1C). Errors in mean T2WI, DWI_(x, y and z)_ and ADC_(x, y and z)_ with a NEX from 3 to 8 were calculated, respectively. Error maps were generated pixel-by-pixel by using the mean images (SI_n_) and the reference images (SI_ref_), where SI_n_ represented signal intensity of T2WI, DWI_(x, y and z)_ and ADC_(x, y and z)_ averaged from n excitations and SI_ref_ represented signal intensity of the reference T2WI, DWI_(x, y and z)_, and ADC_(x, y and z)_, respectively. Error percentage was computed according to the equation of (|SI_n_—SI_ref_ |)/ (SI_ref_) × 100% for T2WI, DWI_(x,y,z)_, and ADC_(x,y,z)_, respectively.

### Statistical analysis

Statistical analysis was performed by using SPSS 12.0 (SPSS, Chicago, III) software. Normality of T2WI, DWI and ADC data was analyzed by using Q-Q plots and Kolmogorov-Smirnov tests. Paired Student *t* test was used for group comparisons of T2WI, DWI, and ADC. Mann-Whitney U test was used for comparing the probabilities of ADC between the healthy group and the radiotherapy group. A *P* value less than 0.05 was considered as statistically significant.

## Results

**Fig 2:**
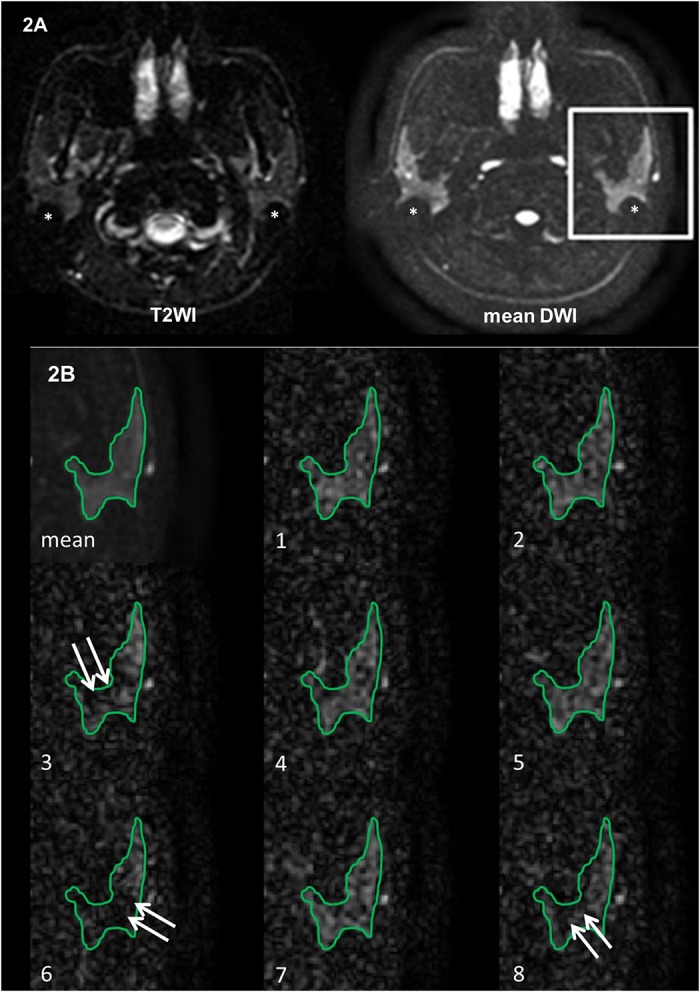
Illustration of transient signal loss on DWI. (A) Mean T2WI and mean DWI show apparent imaging distortion and signal loss (*) on the posterior border of the bilateral parotid glands. (B) Magnified mean DWI and 1^st^ to 8^th^ excitations of DWI of the rectangular ROI encompassing the left parotid gland show patchy areas of signal loss (arrows) in 3^rd^, 6^th^ and 8^th^ excitations.

EPDWI was successfully acquired in all subjects. An example of EPDWI containing parotid glands was displayed in Fig 2. Geometric distortion and concurrent signal loss involving the posterior portion of bilateral parotid glands were obviously seen on both T2WI and mean DWI (Fig 2A). Prominent signal loss involving the left parotid gland was also demonstrated on several excitations of DWI (Fig 2B). Such transient signal loss was neither detected on T2WI, nor on mean DWI. Accordingly, it was necessary to perform an excitation-by-excitation analysis of DWI to catch such transient signal loss. In our study, a total of 464 ROIs (2 ROIs per slice, 5 slices and 8 excitations per subject in 5 subjects plus 2 ROIs per slice, 4 slices and 8 excitations in 1 subject, who had smaller parotid glands) in healthy subjects and 448 ROIs (2 ROIs per slice, 4 slices and 8 excitations per patient in 7 patients) in radiotherapy patients, respectively, were employed for further analysis.

**Fig 3:**
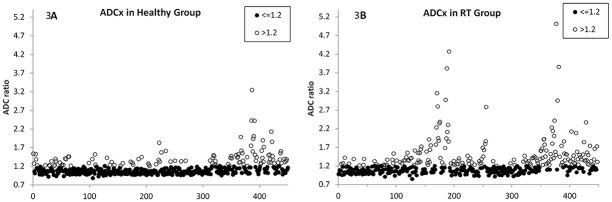
Scatter plots of ADC ratio versus number of all parotid ROIs along x direction in the healthy group (464 ROIs) (3A) and the radiotherapy (RT) group (448 ROIs) (3B). Data are classified into two categories (ADC ratio ≤ 1.2 shown in black circle; ADC ratio > 1.2 shown in white circle). Results along y and z directions were similar to that along x direction (not shown here).

Scatter plots of ADC ratios along the x direction for healthy subjects and radiotherapy patients were shown on Fig 3. While most ROIs had ADC ratios no more than 1.2 (dark circles), some ROIs showed ADC ratios more than 1.2 (white circles). The phenomenon with an ADC ratio more than 1.2 was detected in all participants, especially in some radiotherapy patients. Results along the y and z directions were similar to those along the x direction (not shown here).

**Fig 4:**
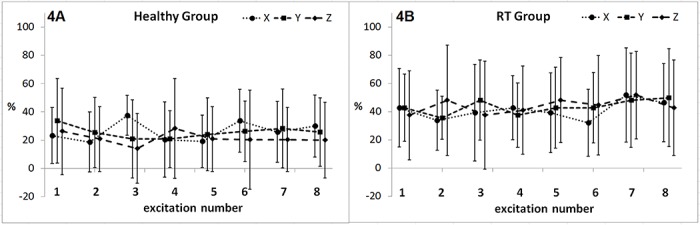
Probability (mean ± standard deviations) of severe overestimation of ADC (ADC ratio > 1.2) at x, y and z directions in 1st to 8th excitations in the healthy group (4A) and the radiotherapy (RT) group (4B). There is no significant difference among 8 excitations or among 3 directions (all *P* > 0.25).

Probabilities of soADC in both healthy subjects and radiotherapy patients were demonstrated on Fig 4. There was no significant difference among three directions of diffusion encoding gradients (*P* = 0.26 to 0.84) and no significant difference among 8 excitations (*P* = 0.57 to 0.99) regarding the probabilities of soADC. It suggested that soADC occurred randomly among 8 excitations and 3 directions of diffusion encoding gradients.

**Fig 5:**
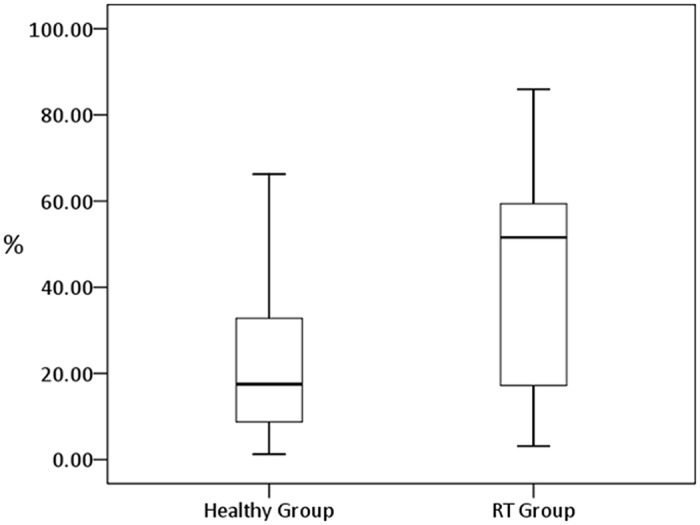
A box-whisker plot shows probability of severe overestimation of ADC (ADC ratio > 1.2) in all parotid ROIs in healthy and radiotherapy (RT) groups.

Box-whisker plots of probabilities of soADC from all ROIs in both healthy and radiotherapy groups were shown on Fig 5. The probability of soADC was significantly higher in the radiotherapy group (42.86%) than the healthy group (24.39%) (*P* < 0.05).

**Fig 6:**
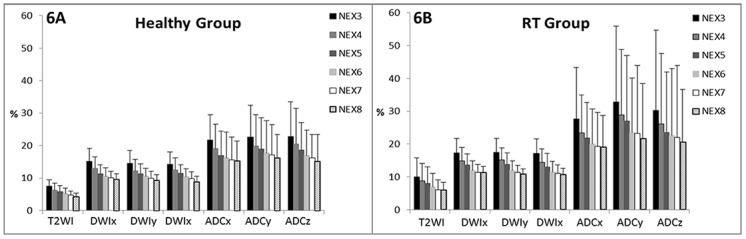
Error percentage (mean and standard deviation) of T2WI, DWI_(x, y and z)_, ADC_(x, y and z)_ with different NEX numbers in healthy group (6A) and radiotherapy (RT) group (6B).

Fig 6 demonstrated error percentages of T2WI, DWIx, DWIy, DWIz, ADCx, ADCy and ADCz in healthy and radiotherapy groups with NEX varying from 3 to 8. It showed that the larger the NEX the lower the error percentage in all images and along all directions of diffusion encoding gradients. In the healthy group, the mean error percentage was 6.32% and 4.37% on T2WI, 13.03% and 9.74% on DWIx, 12.24% and 9.38% on DWIy, 12.52% and 9.00% on DWIz, 19.05% and 15.29% on ADCx, 19.80% and 16.27% on ADCy, 20.45% and 15.17% on ADCz with a NEX of 4 and 8, respectively, (all *P* < 0.001). In the radiotherapy group, the mean error percentage was 8.76% and 6.06% on T2WI, 14.97% and 11.28% on DWIx, 14.37% and 10.76% on DWIy, 15.14% and 10.87% on DWIz, 23.41% and 19.03% on ADCx, 28.95% and 21.68% on ADCy, and 26.17% and 20.58%on ADCz with a NEX number of 4 and 8, respectively, (all *P* < 0.001). The error percentage was smallest on T2WI, followed by DWI and ADC in an increasing order regarding all directions of diffusion encoding gradients and all NEXs. The trends of error percentage regarding the NEX and among T2WI, DWI and ADC remained similar between healthy and radiotherapy groups. Nevertheless, the error percentage was higher in the radiotherapy group than in the healthy group at all numbers of excitations and on all kinds of images (all *P* < 0.001).

## Discussion

Parotid ADC might be altered by diseases including tumor [1, 9] and inflammation [4, 10], radiation [5, 11], gustatory stimulation [12, 13], physiological factors including diffusion, perfusion, and salivary flow [6], histological components such as fat content [7, 14], and technical factors such as strength and number of b values [6], pulse sequences [7, 8], fat saturations, and acceleration factors [7]. Our study further reveals overestimation of ADC in parotid glands on single shot EPDWI due to transient signal loss on DWI (Fig 2B).

The cause of transient signal loss on DWI remains to be determined. One possible cause of transient signal loss on DWI is bulk motion. Bulk motion remains a challenge on diffusion-weighted MR imaging in brain [15, 16], head and neck [17], and liver [18]. It might be due to voluntary or involuntary movement of healthy volunteers and patients. In head and neck, bulk motion might result from vascular pulsation or involuntary motion of subjects. The latter includes swallowing, lip twisting, odontoprisis, mouth breathing, involuntary mouth opening, and jaw clenching [17]. When it causes severe motion artifact that is clearly seen on T2WI or mean DWI, the DWI data can be simply discarded to avoid miscalculation of ADC. On the contrary, when it is not perceptible on T2WI or mean DWI as demonstrated in our study, there is no chance to detect or remedy it in daily practice since the DWI data have been averaged directly. Consequently, the signal loss on DWI unavoidably leads to overestimation of ADC [16]. By using dynamic EPDWI, our study allows us to successfully catch the signal loss on DWI that occurs transiently and randomly among different excitations and different directions of diffusion gradients.

In this study, we introduced an ADC ratio more than 1.2 to represent the occurrence of soADC. This value was chosen because that differences of ADC between malignant and benign parotid tumors [19], between parotid carcinoma and lymphoma [20], and between parotid carcinoma and Warthin’s tumor [20] are all around 20%. When the ADC ratio exceeds 1.2, it would be difficult to differentiate the aforementioned tumor entities. Moreover, the occurrence of soADC also makes the evaluation of ADC change during gustatory stimulation more difficult, since the difference of ADC before and after gustatory stimulation is far less than 20%, i.e. 8.6% in Thoney’s study [12] and 5% in Habermann’s study [13]. Accordingly, it is not appropriate to ignore soADC (Fig 3), which occurs independently to directions of diffusion encoding gradients and randomly among 8 excitations (Fig 4).

Radiation therapy causes histological and physiological changes of parotid glands [21–24]. As a result, salivary flow decreases and xerostomia occurs after radiation exposure of the parotid glands [25, 26]. In the past, DWI has been applied to evaluate the parotid gland function before and after radiotherapy [5, 11]. Zhang et al report that the parotid ADC after radiotherapy is lower than that before radiotherapy [5]. On the other hand, Dirix et al disclose an opposite result, i.e. the parotid ADC after radiotherapy is higher [11]. Our results show a significantly higher probability of soADC in radiotherapy patients (42.86%) than healthy volunteers (24.39%) (*P* < 0.05) (Fig 5). The error percentage in radiotherapy patients is also significantly higher than that in healthy group regarding T2WI, DWI_(x, y and z)_ and ADC_(x, y and z)_ at all NEX (Fig 6). Our results suggest the occurrence of transient signal loss on DWI and overestimation of ADC on radiotherapy patients may overwhelm the radiation effect on parotid ADC measurements. To clarify the influence of transient signal loss of DWI on radiotherapy patients, we have launched a new study to acquire dynamic EPDWI in same group of patients before and after radiotherapy. In addition, whether the higher probability of soADC and the higher estimation error on radiotherapy patients are due to more severe and more frequent bulk motions occurring in radiotherapy patients is still questionable to date. It deserves future work to clarify it by detecting the involuntary bulk motion and the occurrence of signal loss on DWI simultaneously.

By increasing the NEX, our results show that the error is decreased on T2WI, DWI, and ADC images (Fig 6). Our results show that the error percentage is significantly lower with a NEX of 8 as compared to that with a NEX of 4 in T2WI, DWI, and ADC maps. With a NEX increases from 4 to 8, the mean error is significantly reduced by a factor of 30.8% to 30.9% on T2WI, 23.4% to 28.2% on DWI, and 17.8% to 25.8% on ADC maps. Nevertheless, the mean errors are about twice on DWI and 3 times on ADC as compared to that on T2WI. Since DWI (b = 1000 s/mm^2^) differs from T2WI (b = 0 s/mm^2^) only by the strength of diffusion encoding gradients, the higher error on DWI might indirectly reflect the effect of bulk motions encoded by the diffusion probing gradients.

Our study has several limitations to be mentioned. First, only 4 to 5 slices rather than all slices covering the entire parotid glands were used for analysis. We did not use slices on the upper and lower edges of parotid glands in order to avoid the partial volume effect. Second, only four images with highest SNR were averaged to generate reference image for T2WI (b = 0), DWI and ADC maps. We did not use the first four images since signal loss might occur in these images as shown in Fig 2B. Third, the sample size of this study was small. The main purpose of this study was to examine the occurrence of signal loss of DWI on an excitation-by-excitation basis. For this sake, we had a total of 240 excitations in healthy subjects and 224 excitations in radiotherapy patients, which were considered sufficient for our main purpose.

In conclusion, our study discloses that transient signal loss on DWI was successfully detected by dynamic EPDWI. The signal loss on DWI and overestimation of ADC could be partially remedied by increasing the NEX.

## Supporting Information

S1 Dataset Raw data from all regions-of-interest for both healthy subjects and patients was shown, including T2-weighted images, diffusion-weighted images, and apparent diffusion coefficient. (XLSX)
